# Mechanism of 2,3-butanediol stereoisomers formation in a newly isolated *Serratia* sp. T241

**DOI:** 10.1038/srep19257

**Published:** 2016-01-12

**Authors:** Liaoyuan Zhang, Zewang Guo, Jiebo Chen, Quanming Xu, Hui Lin, Kaihui Hu, Xiong Guan, Yaling Shen

**Affiliations:** 1 Key Laboratory of Biopesticide and Chemical Biology, Ministry of Education, College of Life Science, Fujian Agriculture and Forestry University, FuZhou, Fujian province, 350002, PR China; 2 State Key Laboratory of Bioreactor Engineering, New World Institute of Biotechnology, East China University of Science and Technology, Shanghai, 200237, PR China

## Abstract

*Serratia* sp. T241, a newly isolated xylose-utilizing strain, produced three 2,3-butanediol (2,3-BD) stereoisomers. In this study, three 2,3-butanediol dehydrogenases (BDH1-3) and one glycerol dehydrogenase (GDH) involved in 2,3-BD isomers formation by *Serratia* sp. T241 were identified. *In vitro* conversion showed BDH1 and BDH2 could catalyzed (3*S*)-acetoin and (3*R*)-acetoin into (2*S*,3*S*)-2,3-BD and *meso*-2,3-BD, while BDH3 and GDH exhibited the activities from (3*S*)-acetoin and (3*R*)-acetoin to *meso*-2,3-BD and (2*R*,3*R*)-2,3-BD. Four encoding genes were assembled into *E*. *coli* with *budA* (acetolactate decarboxylase) and *budB* (acetolactate synthase), responsible for converting pyruvate into acetoin. *E. coli* expressing *budAB*-*bdh1/2* produced *meso*-2,3-BD and (2*S*,3*S*)-2,3-BD. Correspondingly, (2*R*,3*R*)-2,3-BD and *meso*-2,3-BD were obtained by *E. coli* expressing *budAB*-*bdh3*/*gdh*. These results suggested four enzymes might contribute to 2,3-BD isomers formation. Mutants of four genes were developed in *Serratia* sp. T241. Δ*bdh1* led to reduced concentration of *meso*-2,3-BD and (2*S*,3*S*)-2,3-BD by 97.7% and 87.9%. (2*R*,3*R*)-2,3-BD with a loss of 73.3% was produced by Δ*bdh3*. Enzyme activity assays showed the decrease of 98.4% and 22.4% by Δ*bdh1* and Δ*bdh3* compared with the wild strain. It suggested BDH1 and BDH3 played important roles in 2,3-BD formation, BDH2 and GDH have small effects on 2,3-BD production by *Serratia* sp. T241.

2,3-butanediol (2,3-BD) and its dehydrogenation product, acetoin (AC), are important biobased chemicals that can be produced by biotechnological routes^1^,^2^. 2,3-BD have potential applications in the manufacture of printing inks, perfumes, fumigants, moistening and softening agents, plasticizers, and as a carrier for pharmaceuticals^3^,^4^,^5^,^6^,^7^. While AC is a natural flavor applied widely in food, cosmetics, pharmacy and chemicals synthesis^8^,^9^,^10^.

2,3-BD contains two stereo centers, resulting in three stereo isomers including (2*R*,3*R*)-, *meso*- and (2*S*,3*S*)-forms, and AC exists in two stereoisomeric forms: (3*R*)-acetoin and (3*S*)-acetoin, which are important potential pharmaceutical intermediates^11^,^12^,^13^. All of the isomers of AC and 2,3-BD can be produced by natural strains. However, the stereoisomeric composition of AC and 2,3-BD formed by bacteria differs among strains, such as *P*. *polymyxa* produced (2*R*,3*R*)-2,3-BD and (3*S*/3*R*)-AC with a small amount of *meso*-2,3-BD^14^, and (2*S*,3*S*)-2,3-BD/*meso*-2,3-BD with (3*S*/3*R*)-AC could be obtained by *K*. *pneumoniae*, *K*. *oxytoca* and *S*. *marcescens*^5^,^15^. In previous studies, two models were proposed for explaining 2,3-BD stereoisomers formation. The model proposed by Voloch *et al.* postulates the presence of an acetoin racemase and two acetoin reductases in *K. pneumoniae*. Two acetoin reductases catalyze the reduction of (3*S*)-AC to (2S,3S)-2,3-BD and (3*R*)-AC to *meso*-2,3-BD, respectively, whereas acetoin racemase is responsible for catalyzing the interconversion between (3*S*)-AC and (3*R*)-AC^16^. Ui *et al.* have proposed another model for the formation of 2,3-BD stereoisomers in *K. pneumoniae* which is involved in three butanediol dehydrogenases: *meso*-butanediol dehydrogenase (*R*-acetoin forming), *meso*-butanediol dehydrogenase (*S*-acetoin forming) and (2*S*,3*S*)-2,3-butanediol dehydrogenase. The authors separated the butanediol dehydrogenases individually and determined their stereospecificities. However, the presence of acetoin or butanediol racemase was not confirmed in their experiments^17^. Recent studies show that three key enzymes including α-acetolactate synthase (ALS), α-acetolactate decarboxylase (ALDC), and 2,3-butanediol dehydrogenase (BDH, also called AC/diacetyl reducatse, AR) are involved in the formation of AC and 2,3-BD stereoisomers from pyruvate^18^,^19^,^20^,^21^. First, two molecules of pyruvate condense to yield α-acetolactate and release one molecule of CO_2_ by ALS, then α-acetolactate is decarboxylated into (3*R*)-AC by ALDC^22^,^23^. Meanwhile, α-acetolactate is also readily to undergo nonenzymatic oxidative decarboxylation and form diacetyl (DA) under oxygen supply conditions^24^. Finally, (3*R*)-AC and DA are reduced into 2,3-BD with the corresponding configurations by different BDHs. In addition, reports have shown that glycerol dehydrogenases (GDH) play an important role in the formation of 2,3-BD stereoisomers. A new model have been proposed for the formation of 2,3-BD stereoisomers in *K. pneumoniae* which includes two enzymes (*meso*-BDH and GDH). The *meso*-BDH enzyme catalyzes the stereospecific conversion of (3*R*)-AC to *meso*-2,3-BD and DA to (2*S*,3*S*)-2,3-BD via (3*S*)-AC as an intermediate, whereas GDH shows the abilities in the conversion from (3*R*)-AC to (2*R*,3*R*)-2,3-BD and DA to *meso*-2,3-BD via (3*S*)-AC as an intermediate. The two enzymes contribute to the formation of three 2,3-BD isomers in *K. pneumoniae*^25^. Therefore, the existence of multiple stereospecific dehydrogenase in natural strains is regarded as key factor for the mixed formation of AC and 2,3-BD stereoisomers^11^,^26^.

Several BDHs and GDHs involved in AC and 2,3-BD isomers formation have been identified and characterized in recent studies. In summary, BDHs can be divided into three classes: *meso*-BDH, (2*S*,3*S*)-BDH and (2*R*,3*R*)-BDH. The *meso*-BDHs from *K*. *pneumoniae* and *S*. *marcescens* belonged to the short-chain dehydrogenase/reductase (SDR) superfamily and contributed to *meso*-2,3-BD and (2*S*,3*S*)-2,3-BD production from (3*R*)-AC and (3*S*)-AC respectively^11^,^27^. While the (2*R*,3*R*)-BDHs from *P*. *polymyxa* and *Bacillus subtilis* belonged to the medium-chain dehydrogenase/reductase (MDR) superfamily and converted (3*R*)-AC and (3*S*)-AC into (2*R*,3*R*)-2,3-BD and *meso*-2,3-BD^14^,^28^. In addition, two (2*S*,3*S*)-BDHs from *Rhodococcus erythropolis* and *Brevibacterium saccharolyticum* were reported to possess absolute substrate stereospecificity in the reduction of DA to (2*S*,3*S*)-2,3-BD via (3*S*)-AC^29^,^30^,^31^. Recent studies showed that GDHs in *K. pneumoniae*, *Bacillus licheniformis* and *S. marcescens* played a dual role in glycerol metabolism and 2,3-BD formation^4^,^32^,^33^. *In vitro* conversion showed that GDH could catalyze the conversion from (3*R*)-AC and (3*S*)-AC to (2*R*,3*R*)-2,3-BD and *meso*-2,3-BD, which is similar to (2*R*,3*R*)-BDH. During sugar fermentation process, *K. pneumoniae* produced a mixture of *meso*-2,3-BD and (2*S*,3*S*)-BD. However, three isomers of 2,3-BD were formed simultaneously when glycerol was used as carbon source, which suggested that the formation of 2,3-BD isomers also was influenced by culture conditions^25^,^33^.

*Serratia* sp. T241, a newly isolated 2,3-BD producing strain in our lab, could utilize xylose and glucose as carbon sources to produce 2,3-BD, exhibiting a potential for 2,3-BD production based on lignocellulose-derived sugars. Interestingly, this strain T241 could produce three isomers of 2,3-BD simultaneously during the sugar fermentation process. Usually natural microorganisms produce a mixture of (2*S*,3*S*)-2,3-BD/*meso*-2,3-BD or (2*R*,3*R*)-2,3-BD/*meso*-2,3-BD. It suggested that there might be some differences for the mechanism of AC and 2,3-BD stereoisomers formation between strain T241 and other reported 2,3-BD producing strains. To clarify the differences of 2,3-BD isomers formation between *Serratia* sp. T241 and other 2,3-BD producing strains, a blast search based on *Serratia* sp. genome sequence was carried out using known functional BDHs (*meso*-BDH, (2*S*,3*S*)-BDH and (2*R*,3*R*)-BDH) and GDHs sequences. The obtained putative BDH/GDH genes were selected to perform *in vitro* and *in vivo* experiments. Heterogenous expression and pathway assembly in *E.* coli along with *budA* (acetolactate decarboxylase) and *budB* (acetolactate synthase), responsible for converting pyruvate into acetoin, confirmed that the putative BDH/GDH enzymes showed the abilities in the interconversion between AC and 2,3-BD, which contributed to all the AC and 2,3-BD isomers formation. Furthermore, gene deletion and enzyme assay verified the roles of the genes for AC and 2,3-BD isomers formation in *Serratia* sp. T241.

## Results

### Identification of putative *Serratia* sp. T241 BDH/GDH genes

A search of the literature resulted in identification of three *meso*-BDH genes, three (2*S*,3*S*)-BDH genes, three (2*R*,3*R*)-BDH genes and three GDH genes that were experimentally linked to their corresponding 2,3-BD dehydrogenase/AC reductase activities: *meso*-BDH genes from *S. marcescens* H30^11^, *K. pneumoniae* XJ-Li^34^ and *K. oxytoca* E718^18^; (2*S*,3*S*)-BDH genes from *R. erythropolis*^29^, *B. saccharolyticum*^30^ and *K. pneumoniae* ATCC200721^35^; (2*R*,3*R*)-BDH genes from *P. polymyxa* ATCC12321^14^, *B. subtilis* 168^36^ and *Saccharomyces cerevisiae*^37^; GDH genes from *S. marcescens* H30^32^, *K. pneumoniae* CGMCC1.6366^33^ and *B. licheniformis* 10-1-A^4^. The 16s rDNA sequence of strain T241 shared a high identity of 99% with *Serratia* sp. AS12. So these gene resources were used to search the *Serratia* sp. AS12 protein database by BioEditor local BLASTp function. Four similar genes (GenBank number: AEF50077, AEF51363, AEF51265 and AEF52434) from *Serratia* sp. AS12 were found, and their deduced amino sequences shared high identities of 95%, 64%, 68% and 91% with *meso*-BDH from *S. marcescens* H30, (2*S*,3*S*)-BDH from *R. erythropolis*, (2*R*,3*R*)-BDH from *B. subtilis* 168 and GDH from *S. marcescens* H30. Therefore, these obtained similar genes designated as *bdh1*, *bdh2*, *bdh3*, and *gdh* might play important roles in 2,3-BD isomers formation in *Serratia* sp. T241.

### Stereospecific characteristics of BDH1, BDH2, BDH3 and GDH enzymes

Four BDH/GDH genes were cloned, expressed and purified as described in “Materials and methods” (Fig. S1). The purified enzymes were used to determine the kinetic parameters using AC and 2,3-BD as substrates under their optimal pH conditions. The comparative data of apparent *K*_m_ and *K*_cat_ values for BDH1-3 and GDH from *Serratia* sp. T241 were given in Table 1. BDH1 and BDH2 showed the activities for (3*S*/3*R*)-AC, *meso*-2,3-BD and (2*S*,3*S*)-2,3-BD as substrates. No activity for BDH1 and BDH2 could be measured when (2*R*,3*R*)-2,3-BD was used as a substrate. The *K*_m_ and *K*_cat_ values of BDH1 were 6.64 mM and 35.25 s^−1^ for *meso*-2,3-BD, and 8.25 mM and 0.34 s^−1^ for (2*S*,3*S*)-2,3-BD, respectively. While the *K*_m_ and *K*_cat_ values of BDH2 were 8.84 mM and 2.8 s^−1^ for *meso*-2,3-BD, and 0.39 mM and 17.10 s^−1^ for (2*S*,3*S*)-2,3-BD, respectively. Therefore, BDH1 and BDH2 could be categorized as *meso*-BDH and (2*S*,3*S*)-BDH. Correspondingly, BDH3 and GDH exhibited similar catalytic properties of (2*R*,3*R*)-BDHs and GDHs from other reported 2,3-BD producing strains in the presence of AC and 2,3-BD isomers as substrates. (3*S*/3*R*)-AC, *meso*-2,3-BD and (2*R*,3*R*)-2,3-BD could be reduced or oxidized by BDH3 and GDH with NADH/NAD^+^ as coenzymes. (2*S*,3*S*)-2,3-BD was not a substrate for BDH3 and GDH at all. In addition, GDH also exhibited high catalytic efficiency for glycerol as a substrate (data no shown). So BDH3 and GDH from *Serratia* sp. T241 should be categorized as (2*R*,3*R*)-BDH and GDH.

**Figure 1:**
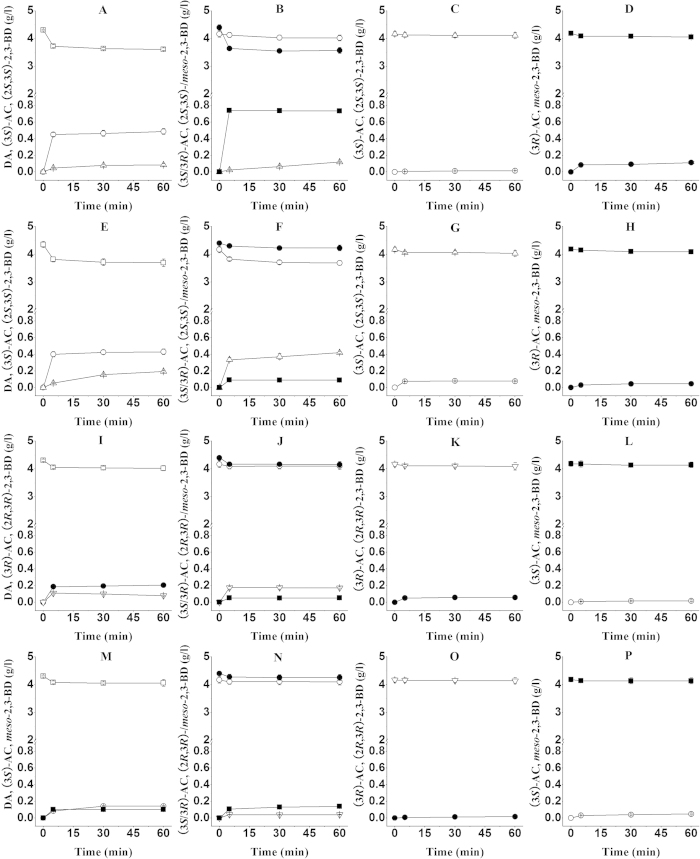
Catalysis reactions of BDH1 (**A–D**), BDH2 (**E-H**), BDH3 (**I–L**) and GDH (**M–P**) enzymes from *Serratia* sp. T241 using DA, AC and 2,3-BD as substrates. DA (empty square), (3*S*)-AC (empty circle), (3*R*)-AC (filled circle), (2*S*,3*S*)-2,3-BD (upright triangle), (2*R*,3*R*)-2,3-BD (inverted triangle), *meso*-2,3-BD (filled square). Error bars represents standard deviation from the mean (n = 3).

The stereospecific characteristics of the four enzymes were investigated by catalytic reactions using DA, (3*S*/3*R*)-AC and 2,3-BD as substrates. The products in these reaction systems were extracted by ethyl acetate and then used to check the enzyme stereospecificity using a GC chromatograph system. Meanwhile, the concentration of the products was determined by calibration curves. Fig. 1 and Fig. S2 demonstrated the four enzymes stereospecificity in the oxidation-reduction processes of the 2,3-BD/AC/DA interconversion. In 2,3-BD oxidation reactions, when (2*S*,3*S*)-2,3-BD and *meso*-2,3-BD were used as the substrates with BDH1 and BDH2, (3*S*)-AC and (3*R*)-AC could be produced respectively (Fig. 1c,d,g,h), whereas BDH3 and GDH showed the abilities in the conversion from (2*R*,3*R*)-2,3-BD and *meso*-2,3-BD to (3*R*)-AC and (3*S*)-AC (Fig. 1k,l,o,p). In reduction reactions, when DA was used as the substrate, (3*S*)-AC and (2*S*,3*S*)-2,3-BD were obtained from DA by BDH1 and BDH2 (Fig. 1a,e). Similar to BDH1 and BDH2, GDH could converted DA into (3*S*)-AC with NADH as coenzyme (Fig. 1m). However, the (3*S*)-AC product from DA by GDH could be further transformed into *meso*-2,3-BD (Fig. 1m). Among the four enzymes, only BDH3 could catalyze DA into (3*R*)-AC, which was further converted into (2*R*,3*R*)-2,3-BD (Fig. 1i). For a racemic mixture of (3*S*/3*R*)-AC reduction reaction, *meso*-2,3-BD and (2*S*,3*S*)-2,3-BD could be formed from (3*S*/3*R*)-AC by BDH1 and BDH2 (Fig. 1b,f). BDH3 and GDH from *Serratia* sp. T241 exhibited high (*R*)-enantioselectivity and catalyzed (3*S*/3*R*)-AC into *meso*-2,3-BD and (2*R*,3*R*)-2,3-BD (Fig. 1j,n).

### Assembly of the BDH/GDH genes with AC operon in *E. coli*

**Figure 2:**
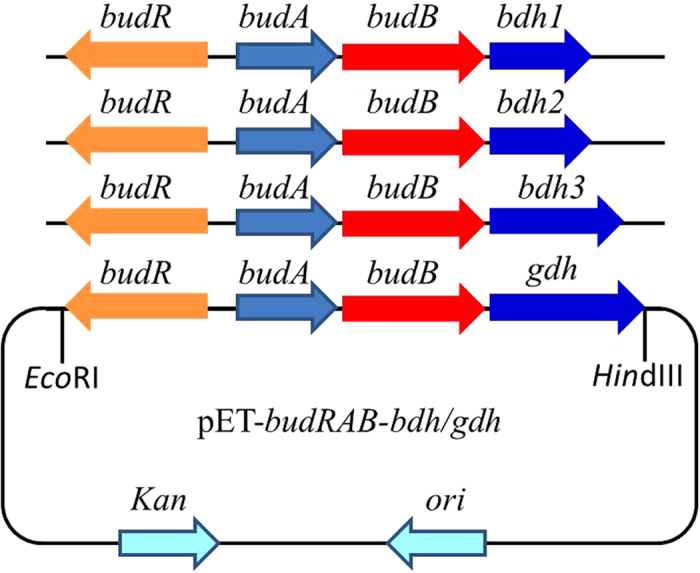
Construction of expression plasmids containing synthetic 2,3-BD operon. The *budR*, *budA* and *budB* from *S. marcescens* H30 encode the transcriptional activator, α-acetolactate decarboxylase and α-acetolactate synthase, respectively. The *bdh1*, *bdh2*, *bdh3* and *gdh* genes from *Serratia* sp. T241 encode the putative 2,3-butanediol dehydrogenase or glycerol dehydrogenase.

**Figure 3:**
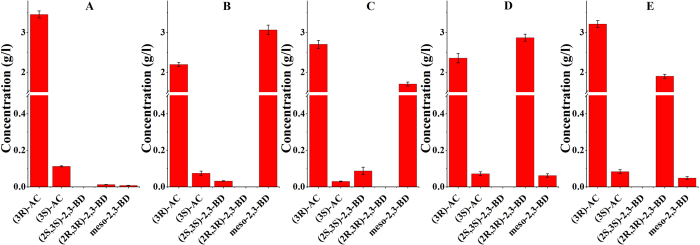
Batch fermentations using recombinant *E.coli* expressing *budRAB* (**A**), *budRAB-bdh1* (**B**), *budRAB-bdh2* (**C**), *budRAB-bdh3* (**D**) and *budRAB-gdh* (**E**). Error bars represents standard deviation from the mean (n = 3).

The roles of BDH1, BDH2, BDH3 and GDH were investigated for the production of AC and 2,3-BD using *E. coli* BL21(DE3) as the host, which has no native AC and 2,3-BD production metabolism. As illustrated in Fig. 2, four genes encoding BDH1, BDH2, BDH3 and GDH from *Serratia* sp. T241 were cloned and assembled along with AC operon from *S. marcescens* H30 into pET28a vector, to generate the plasmids pET-*budRAB-bdh1*, pET-*budRAB-bdh2*, pET-*budRAB-bdh3* and pET-*budRAB-gdh*. All of the plasmids harbored the AC operon (*budRAB*), which is used to produce acetoin from pyruvate. The genes *bdh1*, *bdh2*, *bdh3* and *gdh* were expressed for evaluating the conversion of AC to 2,3-BD. The recombinant *E. coli* strains were subjected to batch fermentation using LB medium with 10 g/l glucose and *E. coli* carrying pET-*budRAB* was used as control. The results were given in Fig. 3. The control strain mainly produced 3.46 g/l of (3*R*)-AC and 0.11 g/l of (3*S*)-AC (Fig. 3a). In addition, a trace amount of (2*R*,3*R*)-2,3-BD and *meso*-2,3-BD could be detected in the broth by the control strain. As shown in Fig. 3b–e, all the strains carrying pET-*budRAB-bdh1*, pET-*budRAB-bdh2*, pET-*budRAB-bdh3* and pET-*budRAB-gdh* produced (3*R*)-AC in high amounts. *meso*-2,3-BD was also produced by all the strains but in much lower amounts for the strains containing *bdh3* and *gdh* genes. The strains containing bdh1 and bdh2 genes formed (2*S*,3*S*)-2,3BD which was absent from the strains containing *bdh3* and *gdh* genes. Instead the strains containing *bdh3* and *gdh* genes produced high amounts of (2*R*,3*R*)-2,3BD, which was totally absent in the other two strains.

### Metabolic characteristics of *Serratia* sp. T241 Δbdh1, Δbdh2, Δbdh3 and Δgdh

**Figure 4:**
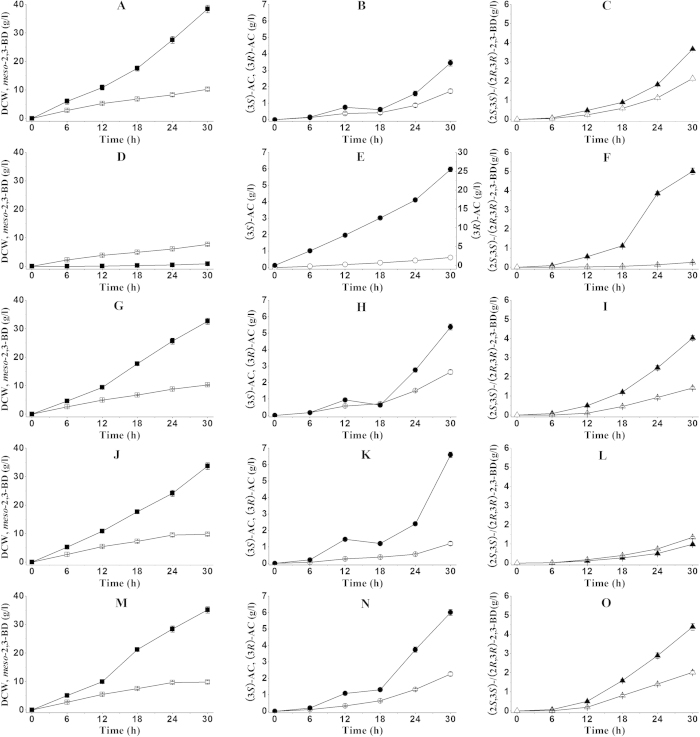
Batch fermentations using *Serratia* sp. T241 (**A–C**) and its mutants including Δbdh1 (**D–F**), Δbdh2 (**G–I**), Δbdh3 (**J–L**) and Δgdh (**M–O**). DCW (empty square), *meso*-2,3-BD (filled square), (3*S*)-AC (empty circle), (3*R*)-AC (filled circle), (2*S*,3*S*)-2,3-BD (empty triangle) and (2*R*,3*R*)-2,3-BD (filled triangle). Error bars represents standard deviation from the mean (n = 3).

*Serratia* sp. T241 Δbdh1, *Serratia* sp. T241 Δbdh2, *Serratia* sp. T241 Δbdh3 and *Serratia* sp. T241 Δgdh were constructed as described in “Materials and methods”. *In vitro* conversion showed that BDH1, BDH2, BDH3 and GDH have the abilities in the interconversion between AC and 2,3-BD. However, the roles of the four genes for 2,3-BD isomers formation in *Serratia* sp. T241 still remained unclear. Whether all the four enzymes regulated AC and 2,3-BD isomers formation by *Serratia* sp. T241 or not was unknown. So the four mutants and the wild strain were cultured in fermentation medium to investigate their metabolic characteristics. The cultures were carried out in 250 ml flask with 50 ml fresh fermentation medium, and the products in the broth were analyzed and quantified by GC system. The results were shown in Fig. 4. The wild strain mainly produced *meso*-2,3-BD of 38.43 g/l with the dry cell weight (DCW) of 10.25 g/l at 30 h. Low levels of (3*S*)-AC, (3*R*)-AC, (2*S*,3*S*)-2,3-BD and (2*R*,3*R*)-2,3-BD were produced at concentrations of 1.73, 3.45, 2.14 and 3.67 g/l respectively (Fig. 4a–c). The cell growth of *Serratia* sp. T241 Δbdh1 was slower than that of the wild strain, and the DCW of 7.7 g/l was obtained at 30h. Deletion of *bdh1* gene in *Serratia* sp. T241 resulted in significant decrease of *meso*-2,3-BD, (3*S*)-AC and (2*S*,3*S*)-2,3-BD, only 0.87 g/l *meso*-2,3-BD, 0.61 g/l (3*S*)-AC and 0.26 g/l (2*S*,3*S*)-2,3-BD was obtained by *Serratia* sp. T241 Δbdh1 at 30 h (Fig. 4d–f). In contrast, higher concentrations of (3*R*)-AC (25.50 g/l) and (2*R*,3*R*)-2,3-BD (5.02 g/l) was accumulated by *Serratia* sp. T241 Δbdh1 (Fig. 4e,f). *Serratia* sp. T241 Δbdh2, *Serratia* sp. T241 Δbdh3 and *Serratia* sp. T241 Δgdh still produced a large amount of *meso*-2,3-BD at concentration of 32.64, 33.72 and 35.17 g/l respectively (Fig. 4g,j,m). A similar growth curve of *Serratia* sp. T241 Δbdh2, *Serratia* sp. T241 Δbdh3, *Serratia* sp. T241 Δgdh and the wild strain was observed (Fig. 4a,d,g,j,m). For *Serratia* sp. T241 Δbdh2, the concentrations of (3*S*)-AC, (3*R*)-AC and (2*R*,3*R*)-2,3-BD were 2.64, 5.38 and 4.03 g/l (Fig. 4h,i), which were higher in comparison to those of the wild strain. While the concentration of (2*S*,3*S*)-2,3-BD with 1.43 g/l showed somewhat lower than that of the wild strain (Fig. 4i). During the batch fermentation process, *bdh3* gene deletion led to accumulation of (3*R*)-AC (6.60 g/l) and decrease of (2*R*,3*R*)-2,3-BD (0.98 g/l) obviously (Fig. 4k,l). Similar to *Serratia* sp. T241 Δbdh3, 6.01 g/l of (3*R*)-AC could be produced by *Serratia* sp. T241 Δgdh (Fig. 4n). However, *Serratia* sp. T241 Δgdh still produced 4.40 g/l of (2*R*,3*R*)-2,3-BD (Fig. 4o). Correspondingly, the enzyme activities of the four mutants and the wild strain during the fermentation process also were measured, and the results were shown in Table 2. The wild strain exhibited higher activity of 15.61 U/mg for (3*S*/3*R*)-AC as a substrate. The activities in *Serratia* sp. T241 Δbdh2, *Serratia* sp. T241 Δbdh3 and *Serratia* sp. T241 Δgdh were 13.54, 12.11 and 13.43 U/mg, which still remained relatively high activities, whereas the enzyme activity of only 0.25 U/mg in *Serratia* sp. T241 Δbdh1 was observed.

## Discussion

2,3-BD and its dehydrogenation product, AC, could be produced by several natural strains such as *K. pneumoniae*^9^, *K. oxytoca*^10^, *S. marcescens*^5^,^11^ and *B. polymyxa*^12^. However, these natural strains produce a mixture of 2,3-BD and AC, which limits their applications. So elucidation of the mechanism for 2,3-BD isomers formation and development of engineered strains for single configuration production of 2,3-BD are required. Previous studies showed that (3*R*)-AC as main intermediate product was produced from pyruvate by ALS and ALDC enzymes, whereas low level of (3*S*)-AC was obtained from DA, which was formed by a nonenzymatic oxidative decarboxylation of α-acetolactate^19^. BDH is a reversible enzyme involved in the last step from AC to 2,3-BD^14^. Recent studies showed that multiple dehdyrogenases could carry out the conversion from AC to 2,3-BD such as (2*S*,3*S*)-BDH, (2*R*,3*R*)-BDH, *meso*-BDH and GDH, which resulted in different configuration formation of 2,3-BD^4^,^13^,^26^. Therefore, the catalytic efficiency of dehydrogenases and the existence of multiple dehydrogenases in 2,3-BD producing strain were regarded as the key factors for 2,3-BD isomers formation.

*Serratia* sp. T241, a newly isolated xylose-utlizing strain, could produce high concentration of 2,3-BD with three configurations. In this study, four genes (*bdh1*, *bdh2*, *bdh3* and *gdh*) involved in AC and 2,3-BD isomers formation in *Serratia* sp. T241 were identified. This is the first report that four enzymes played roles in 2,3-BD isomers production in one strain. The sequencing results showed the *bdh1*, *bdh2*, *bdh3* and *gdh* genes from *Serratia* sp. T241 shared high identities of 88%, 64%, 64% and 86% with *meso*-BDH from *S. marcescens* H30, (2*S*,3*S*)-BDH from *R. erythropolis*, (2*R*,3*R*)-BDH from *B. subtilis* 168 and GDH from *S. marcescens* H30, implying that the four enzymes might contribute to 2,3-BD isomers formation in *Serratia* sp. T241. The four genes encoding the BDH1, BDH2, BDH3 and GDH enzymes was cloned and expressed in *E. coli* BL21(DE3), purified and characterized. All the four purified enzymes exhibited the activities for AC and 2,3-BD as substrates. The main differences occurred in 2,3-BD oxidation reaction. (2*S*,3*S*)-2,3-BD and *meso*-2,3-BD were the substrates of BDH1 and BDH2, which showed no activity for (2*R*,3*R*)-2,3-BD. While BDH3 and GDH showed the activities for (2*R*,3*R*)-2,3-BD and *meso*-2,3-BD as substrates, (2*S*,3*S*)-2,3-BD was not the substrate for BDH3 and GDH at all. According to their *K*_m_ and *K*_cat_ values, the four enzymes (BDH1, BDH2, BDH3 and GDH) should be categorized as *meso*-BDH, (2*S*,3*S*)-BDH, (2*R*,3*R*)-BDH and GDH. BDH3 was the first reported (2*R*,3*R*)-BDH from Gram-negative strain.

**Figure 5:**
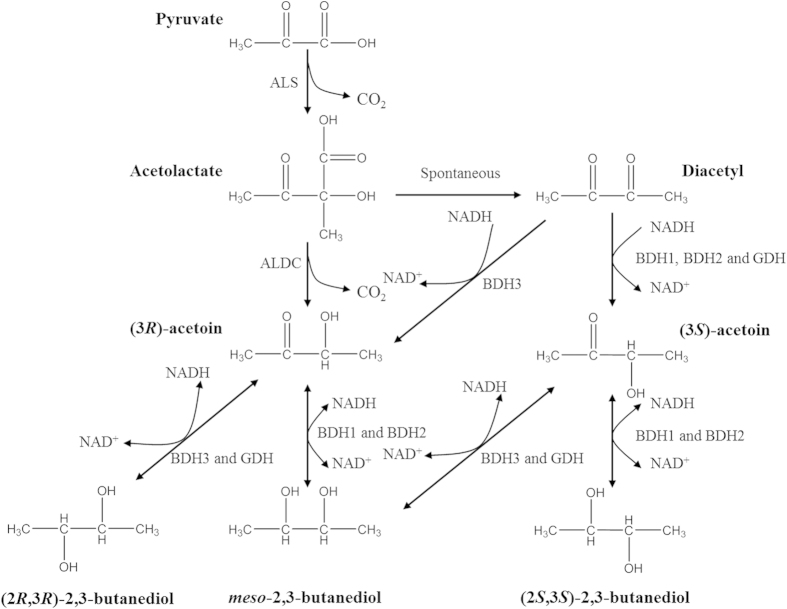
2,3-Butanediol biosynthesis pathway and mechanism of 2,3-butanediol stereoisomer formation in *Serratia* sp. T241. ALS (acetolactate synthase), ALDC (acetolactate decarboxylase), BDH1 (*meso*-2,3-butanediol dehydrogenase), BDH2, ((2*S*,3*S*)-2,3-butanediol dehydrogenase), BDH3 ((2*R*,3*R*)-2,3-butanediol dehydrogenase), GDH (glycerol dehydrogenase).

Furthermore, *in vitro* conversion and *in vivo* assemble of 2,3-BD pathway in *E. coli* revealed that the four enzymes contributed to *meso*-2,3-BD production in *Serratia sp*. T241. (2*S*,3*S*)-2,3-BD was obtained from (3*S*)-AC by BDH1 and BDH2, whereas BDH3 and GDH led to (2*R*,3*R*)-2,3-BD formation from (3*R*)-AC. The detailed model of AC and 2,3-BD isomers formation by *Serratia* sp. T241 could be inferred from the data of stereospecificity in catalytic reaction by the four purified enzymes (Fig. 1 and Fig. S2). As shown in Fig. 5, (3R)-AC was produced from pyruvate via α-acetolacetate by ALS and ALDC, and DA was formed by a non-enzymatic oxidation decarboxylation of α-acetolactate. In *Serratia* sp. T241, (3*S*)-AC production from DA was afforded by BDH1, BDH2 and GDH. The features of BDH1 and BDH2 were similar to reported *meso*-BDH and (2*S*,3*S*)-BDH from *K*. *pneumoniae* and *R. erythropolis* respectively^27^,^29^. However, the GDH enzymes in previous studies showed no activity using DA as a substrate except that GDH from *S. marcescens* H30 which could converted DA into (3*S*)-AC and exhibited the same catalytic property with the GDH from *Serratia* sp. T241^25^,^32^. DA also was converted into (3*R*)-AC due to the existence of BDH3 in *Serratia* sp. T241. BDH1 and BDH2 exhibited high (*S*)-enantioselectivity for AC as substrate and catalyzed (3*S*)-AC and (3*R*)-AC into (2*S*,3*S*)-2,3-BD and *meso*-2,3-BD, respectively. In contrast, high (*R*)-enantioselectivity of BDH3 and GDH led to (2*R*,3*R*)-2,3-BD and *meso*-2,3-BD production from (3*R*)-AC and (3*S*)-AC. Similar to reported BDH/GDH from other strains, the four enzymes showed the abilities in the conversion from 2,3-BD to AC, and the corresponding configuration of AC could be obtained. It suggested that BDH1, BDH2, BDH3 and GDH were four reversible enzymes for the interconversion between AC and 2,3-BD. In addition, no DA could be detected from any form of 2,3-BD and AC, which showed that the conversions in catalytic reactions by the four enzymes were irreversible between DA and AC. In previous studies, the model of 2,3-BD stereoisomers formation in *K*. *pneumoniae* has been revealed^25^. In this model, two enzymes *meso*-BDH and GDH were identified, and contributed to three configurations of 2,3-BD production. The GDH enzyme in *K*. *pneumoniae* was responsible for (2*R*,3*R*)-2,3-BD and *meso*-2,3-BD formation from (3*R*)-AC and (3*S*)-AC, while (2*S*,3*S*)-2,3-BD and *meso*-2,3-BD were produced from (3*S*)-AC and (3*R*)-AC by *meso*-BDH. *K*. *pneumoniae* deficient in one of the two enzymes only produced two configuration of 2,3-BD^25^,^33^. For *Serratia* sp. T241, four enzymes including *meso*-BDH, (2*S*,3*S*)-BDH, (2*R*,3*R*)-BDH and GDH were identified and contributed to three configuration of 2,3-BD formation, which showed that the model of 2,3-BD stereoisomers formation in *Serratia* sp. T241 was more complicated than that of *K*. *pneumoniae*. According the catalytic properties of four enzymes, inactivating one of the genes encoding the four enzymes in *Serratia* sp. T241 still forms three configuration of 2,3-BD. The significance of such a complicated mechanism for physiological metabolism of *Serratia* sp. T241 still remain unknown, which needs further study in future.

Though *in vitro* conversion and *in vivo* assemble of 2,3-BD pathway in *E. coli* exhibited the catalytic properties and stereospecificity of four enzymes for DA, AC and 2,3-BD isomers as substrates, the expression level of four enzymes in *Serratia* sp. T241 during the fermentation process might be quite different. Therefore, four mutants deficient in BDH1, BDH2, BDH3 and GDH were constructed for investigating their roles in 2,3-BD isomers formation by *Serratia* sp. T241. As shown in Table 2, the enzyme activity loss of 98.4% could be observed in *Serratia* sp. T241 Δbdh1, resulting in an obvious decrease of 2,3-BD yield, especially *meso*-2,3-BD with the loss of 97.7% when compared with the wild strain. A higher concentration of (3*R*)-AC (25.50 g/l) could be accumulated due to the deficiency of BDH1. In addition, deletion of *bdh1* gene also led to low level of (2*S*,3*S*)-2,3-BD with the loss of 87.9%. So BDH1 played a vital role in 2,3-BD production in *Serratia* sp. T241. In contrast, slightly decrease of the enzyme activities was detected in the mutants of Δ*bdh2*, Δ*bdh3* and Δ*gdh*, which still produced a large amount of *meso*-2,3-BD. However, the yields of *meso*-2,3-BD by the three mutants were slightly less than that of the wild strain, implying that BDH2, BDH3 and GDH might also contributed to *meso*-2,3-BD formation. During the fermentation process, low level of (2*R*,3*R*)-2,3-BD with the decrease of 73.3% was produced by *Serratia* sp. T241 Δ*bdh3*. Therefore, BDH3 might played an important role in (2*R*,3*R*)-2,3-BD production in *Serratia* sp. T241. Low concentration of (2*R*,3*R*)-2,3-BD in *Serratia* sp. T241 Δ*bdh3* was produced partially due to the contribution of GDH. Similarly, BDH2 also contributed to (2*S*,3*S*)-2,3-BD production since a small amount of (2*S*,3*S*)-2,3-BD could be detected in *Serratia* sp. T241 Δ*bdh1*. These results showed that the four enzymes contributed to AC and 2,3-BD stereoisomers formation in *Serratia* sp. T241 during the fermentation process. The ratio of AC and 2,3-BD stereoisomers in the broth by *Serratia* sp. T241 partially depended on the catalytic efficiencies and expression level of the four enzymes. Considering the discovery of the four enzymes from the blast search based on the reported gene sequences, other dehydrogenase in *Serratia* sp. catalyzing the conversion from AC to 2,3-BD remained unknown. So it is necessary that development of the mutant deficient in all the four genes in future confirm 2,3-BD accumulation or not.

In a conclusion, four genes involved in 2,3-BD isomers formation by *Serratia* sp. T241 were identified. BDH1, BDH2, BDH3 and GDH encoded by the four genes were categorized as *meso*-2,3-BD dehydrogenase, (2*S*,3*S*)-2,3-BD dehydrogenase, (2*R*,3*R*)-2,3-BD dehydrogenase and glycerol dehydrogenase, and contributed to 2,3-BD isomers formation. During the fermentation process, BDH1 and BDH3 played main roles in 2,3-BD production, while BDH2 and GDH only had small effect on 2,3-BD production in *Serratia* sp. T241.

## Methods

### Enzyme and chemicals

Restriction enzymes and DNA Polymerase High Fidelity were obtained from TaKaRa Biotech (Dalian, China). T4 DNA ligase was obtained from New England Biolabs (Beijing, China). DNA and protein marker were purchased from Tiangen Biotech (Shanghai, China). Bacterial Genomic DNA Miniprep Kit was purchased from BIODEV Corp. (Beijing, China). The primers were synthesized in SBSbio (Shanghai, China) and listed in Table 3. (3S/3R)-AC, (2*S*,3*S*)-2,3-BD (97.0%), (2*R*,3*R*)-2,3-BD (97.0%), *meso*-2,3-BD (99.0%) were obtained from Sigma-Aldrich (Shanghai, China). All other chemicals were of analytical grade and commercially available.

### Bacterial strains, plasmids and bacterial growth condition

The strains and plasmids used in this study are listed in Table 4. *Escherichia coli* DH5α and BL21(DE3) as the cloning and expression hosts were grown at 37 °C. The pET28a vector was used for enzyme expression and the suicide vector pUTKm1 was used for gene knockout in *Serratia* sp. T241. Luria-Bertani (LB) medium was used for cultivation of *E. coli* and *Serratia* sp. T241. LB medium with 10 g/l glucose was used to cultivate *E. coli* containing the recombinant 2,3-BD pathway. Antibiotics were added in the following amounts (per ml) if necessary: 50 μg kanamycin or 50 μg ampicillin.

*Serratia* sp. T241 was maintained on agar slants in LB medium. The slants were incubated at 30 °C, and fully grown slants were stored at 4 °C. For seed preparation, a full loop of cells from a fully grown slant was inoculated into 30 ml of the seed medium in 250 ml flasks and incubated on a rotary shaker for 12 h at 30 °C and 180 rpm. The seed medium^5^ contained the following (per liter) at pH 7.2: glucose 10 g, yeast extract 1 g, peptone 2 g, (NH_4_)_2_SO_4_ 6 g, KH_2_PO_4_ 10 g, NaCl 0.5 g, MgSO_4_ 0.5 g. Subsequently, seed culture (5%, v/v) was inoculated into the fermentation medium which consisted of (per liter): glucose 90 g, yeast extract 15 g, sodium acetate 4 g, NH_4_H_2_PO_4_ 1 g, MgSO_4_ 0.3 g, and MnSO_4_ 0.1 g at pH 7.2. The fermentation experiments were conducted in 250 ml flasks containing 50 ml fresh fermentation medium for 30 h at 30 °C and 180 rpm. All flask experiments were performed in parallel triplicate tests. The data shown are the average of three fermentation runs with standard deviation.

### Identification of putative *Serratia* sp. T241 BDH/GDH genes

Blast searches of *Serratia* sp. AS12 local protein database developed by BioEditor software were carried out using known functional BDHs and GDHs. *meso*-BDH and GDH sequences are from *S. marcescens* H30^11^,^32^, while (2*S*,3*S*)-BDH and (2*R*,3*R*)-BDH sequences are from *R. erythropolis*^29^ and *B. subtilis*^36^ respectively. The putative genes with high scores were collected as candidate genes for further experiments.

### Construction of *E. coli* BL21(DE3)/*BDH* and *E. coli* BL21(DE3)/*GDH*

The encoding sequences of the putative BDH and GDH genes were amplified by PCR with the genomic DNA of *Serratia* sp. T241 as template using the primers (Table 3), which contained the *Eco*RI and *Hin*dIII restriction sites respectively. The amplified products were ligated into the vector pET-28a at *Eco*RI and *Hin*dIII sites to generate the recombinant plasmids. The recombinant plasmids were transformed into *E. coli* BL21(DE3) for commercial sequencing and protein expression.

### Enzyme preparation, assay, and enzymatic reactions

The recombinant strains were cultured at 37 °C in a 250-ml flask containing 50 ml LB medium with kanamycin (50 μg/ml), and expression was induced at a 2.5 h culture (about 0.6 OD_600_) with 0.5 mM isopropyl-beta-D-thiogalactopyranoside (IPTG). Cells were harvested by centrifugation after 6 h, and cell lysate was prepared by sonication in an ice bath. The homogenate was centrifuged at 13,000 × g for 10 min to remove the debris^11^. The soluble fraction was subjected to purification under non-denaturing conditions with Ni-affinity chromatography using a Histrap HP column according the purification protocol (GE Healthcare, USA). The eluate from the column was pooled and desalted by a Hitrap desalting column (GE Healthcare, USA).

Enzyme activity was determined spectrophotometrically by measuring the changes in absorbance at 340 nm and 40 °C corresponding to the oxidation of NADH or the reduction of NAD^+^. The reaction mixtures containing 50 mM potassium phosphate buffer, 0.2 mM NAD^+^ for the oxidation reactions or 50 mM potassium phosphate buffer, 0.2 mM NADH for the reduction reactions were incubated at 40 °C for 5 min. After adding 10 μl of approximately diluted purified enzyme solution, the reaction was started by the addition of the substrates. One unit of BDH activity was defined as the amount of enzyme required to reduce 1 μmol of NAD(H) in one minute^11^. All enzyme activities were determined in triplicate. The protein concentrations of all samples were determined using the Bradford method, and bovine serum albumin served as the standard protein.

The enzymatic reactions were carried out similar to the assay method, except that the substrate and coenzyme concentrations were high. For the oxidation processes, a mixture containing 50 mM *meso*-2,3-BD/(2*S*,3*S*)-2,3-BD/(2*R*,3*R*)-2,3-BD, 4 mM NAD^+^, 50 mM potassium phosphate buffer and 20 μg of purified enzyme in a final volume of 1 ml was incubated at 40 °C for 1 h. The reduction processes were carried out in 1 ml reaction system containing 50 mM DA or 100 mM (3*S*/3*R*)-AC, 4 mM NADH, 50 mM of potassium phosphate buffer and 20 μg of purified enzyme at 40 °C for 1 h. The products in these reaction systems were extracted by ethyl acetate and then used to check the enzyme stereospecificity using a GC chromatograph system^32^.

### Assembly of the putative BDH/GDH genes with AC operon in *E. coli*

The putative BDH/GDH genes involved in 2,3-BD synthesis in *Serratia* sp. T241 and AC operon (*budRAB*) from *S. marcescens* H30 were amplified using the primers listed in Table 3, and assembled as illustrated in Fig. 2. First, the putative genes from *Serratia* sp. T241 and AC operon from *S. marcescens* H30 were amplified by PCR using the genomic DNA of strain T241 and H30 as templates. The PCR products of the putative genes with RBS sequences were assembly into the downstream region of AC operon respectively by overlap extension PCR using the corresponding primers (Table 3). These overlapping PCR fragments were cloned into pET28a vector between the *Eco*RI and *Hin*dIII sites to generate the recombinant 2,3-BD pathway in *E. coli*^18^.

### Development of *Serratia* sp. mutant strains

Two DNA fragments (about 800 bp) from upstream sequence and downstream sequence of the putative gene with overlapping ends were amplified from *Serratia* sp. T241 using the primers listed in Table 3. The two fragments are then combined by overlapping PCR, generating an in-frame deletion construct of the putative gene. The overlapping PCR fragment was digested and cloned into the suicide vector pUTKm1 and transformed into *E. coli* S17-1 λ_pir_ for conjugation with *Serratia* sp. T241. LB medium agar plate containing 50 μg/ml kanamycin was used to screen the single crossover strains. The obtained single crossover strains were confirmed by PCR method. Then, one single crossover strain was grown in LB broth overnight, and plated onto LB medium agar plate. The double crossover strains were screened through kanamycin resistance phenotype^38^. The kanamycin-sensitive colonies were verified by PCR using the primers listed in Table 3.

The other putative genes mutants of *Serratia* sp. T241 were constructed using the same method. The obtained mutants of *Serratia* sp. T241 were stored in a glycerol suspension at −80 °C for further experiments.

### Analytical methods

The biomass concentration was determined by the optical density (OD) measured at 600 nm in a spectrophotometer (UV-1800, MAPADA) and correlated with dry cell weight (DCW)^11^.

The products in the broth were extracted by ethyl acetate with the addition of isopropanol as internal standard and then quantified using a gas chromatograph system (Agilent GC9860) with equipped with a chiral column (Supelco β-DEX™ 120, 30-m length, 0.25-mm inner diameter). The operation conditions were as follows: N_2_ was used as the carrier gas at flow rate of 1.2 ml/min; the injector temperature and the detector temperature were 215 and 245 °C, respectively; and the column temperature was maintained at 50 °C for 1.5 min, then raised to 180 °C at a rate of 15 °C/min. The concentration of the products was determined by calibration curves^11^,^32^.

## Additional Information

**How to cite this article**: Zhang, L. *et al.* Mechanism of 2,3-butanediol stereoisomers formation in a newly isolated *Serratia* sp. T241. *Sci. Rep.* **6**, 19257; doi: 10.1038/srep19257 (2016).

## Supplementary Material

Supplementary Information

## Figures and Tables

**Table 1:** Kinetic parameters of BDH1, BDH2, BDH3 and GDH from *Serratia* sp. T241 for (3*S*/3*R*)-AC, *meso*-2,3-BD, (2*R*,3*R*)-2,3-BD, and (2*S*,3*S*)-2,3-BD^a^.

Enzymes/substrates		(3*S*/3*R*)-AC	*meso*-2,3-BD	(2*R*,3*R*)-2,3-BD	(2*S*,3*S*)-2,3-BD
BDH1	*K*_m_ (mM)	3.95 ± 0.21	6.64 ± 0.14	ND^b^	8.25 ± 0.11
	*K*_cat_ (s^−1^)	228.19 ± 8.07	35.25 ± 1.91	ND	0.34 ± 0.01
BDH2	*K*_m_ (mM)	0.30 ± 0.03	8.84 ± 0.17	ND	0.39 ± 0.03
	*K*_cat_ (s^−1^)	80.22 ± 3.15	2.80 ± 0.04	ND	17.10 ± 0.45
BDH3	*K*_m_ (mM)	0.18 ± 0.01	0.30 ± 0.02	0.21 ± 0.01	ND
	*K*_cat_ (s^−1^)	18.84 ± 0.78	2.44 ± 0.11	2.91 ± 0.10	ND
GDH	*K*_m_ (mM)	0.29 ± 0.02	5.70 ± 0.15	79.85 ± 1.34	ND
	*K*_cat_ (s^−1^)	1.34 ± 0.05	0.33 ± 0.02	0.15 ± 0.01	ND

^a^Assay conditions: the reduction reactions were prepared with 50 mM potassium phosphate buffer (pH 5.0 for BDH1, pH 8.0 for BDH2, BDH3 and GDH) and 0.2 mM NADH, the oxidation reactions were prepared with 50 mM potassium phosphate buffer (pH 8.0) and 0.2 mM NAD^+^. Various concentration of (3*S*/3*R*)-AC, *meso*-2,3-BD, (2*R*,3*R*)-2,3-BD, and (2*S*,3*S*)-2,3-BD range from 10 μM to 20 mM at 40 °C. The *K*_m_ and *K*_cat_ values were obtained by non-linear fitting with the Michaelis-Menten equation.

^b^ND: not detected.

± represents standard deviation from the mean (n = 3).

**Table 2:** Enzyme activity assays of the four gene mutants and the wild strain^a^.

Strains	Enzyme activity (U/mg)	Loss of enzyme activity (%)
*Serratia* sp. T241	15.61 ± 0.27	0
*Serratia* sp. T241 Δbdh1	0.25 ± 0.01	98.4
*Serratia* sp. T241 Δbdh2	13.54 ± 0.43	13.3
*Serratia* sp. T241 Δbdh3	12.11 ± 0.07	22.4
*Serratia* sp. T241 Δgdh	13.43 ± 0.15	14.0

^a^Assay conditions: 50 mM potassium phosphate buffer (pH 7.0), 50 mM (3*S*/3*R*)-AC, 0.2 mM NADH at 40 °C.

± represents standard deviation from the mean (n = 3).

**Table 3:** The primers used in this study.

Primers	Sequence (5′-3′)	
Primers for genes expression		
BDH1F1	CGGAATTCATGCGTTTCGACAATAAAGTGGT	*Eco*RI
BDH1R1	GACAAGCTTTCAAACGATCTTCGGTTGACC	*Hin*dIII
BDH2F1	TCCGAATTCATGTCGACAGGTTTGAACGG	*Eco*RI
BDH2R1	GACAAGCTTTTAGCGATAAACCAGCCCGC	*Hin*dIII
BDH3F1	TCCGAATTCATGGTTAATTTCAAGGGG	*Eco*RI
BDH3R1	GACAAGCTTCCTGCGGGACATTTTACT	*Hin*dIII
GDHF1	CGGAATTCATGTTGAGAATCATCCAGTC	*Eco*RI
GDHR1	GACAAGCTTTCAGCGTTGGTGTTGTTGCAG	*Hin*dIII
Primers for 2,3-BD pathway		
budRABF	CGGAATTCTTACCCCCAACTGGGCGGCT	*Eco*RI
BudRABR	CATATGTATATCCTCCTTATTAAATCATCTGGCTGAAGT	RBS squence
BDH1F2	GATTTAATAAGGAGGATATACATATGCGTTTCGACAATAAAGT	RBS squence
BDH1R2	GACAAGCTTTCAAACGATCTTCGGTTGACC	*Hin*dIII
BDH2F2	GATTTAATAAGGAGGATATACATATGTCGACAGGTTTGAACGG	RBS squence
BDH2R2	GACAAGCTTTTAGCGATAAACCAGCCCGC	*Hin*dIII
BDH3F2	GATTTAATAAGGAGGATATACATATGGTTAATTTCAAGGGG	RBS squence
BDH3R2	GACAAGCTTCCTGCGGGACATTTTACT	*Hin*dIII
GDHF2	GATTTAATAAGGAGGATATACATATGTTGAGAATCATCCAGTC	RBS squence
GDHR2	GACAAGCTTTCAGCGTTGGTGTTGTTGCAG	*Hin*dIII
Primers for gene deletion		
BDH1UF	GGGGTACCAGCCGAAGTGTAACCTGAA	*Kpn*I
BDH1UR	TTTCACCAGGCTCGGGCACAGGGCAAGAGCCAGTCAA	
BDH1DF	TTGACTGGCTCTTGCCCTGTGCCCGAGCCTGGTGAAA	
BDH1DR	GTGAGTACTGGTGAAGGCTCGCTATGTG	*Sca*I
BDH2UF	GGGGTACCCGCCAGCCTCTACAACGATC	*Kpn*I
BDH2UR	CGGTTGAGTAAGCACCTAAATCCCGTATTGTCCTACTGAT	
BDH2DF	ATCAGTAGGACAATACGGGATTTAGGTGCTTACTCAACCG	
BDH2DR	GTGAGTACTTCCTGCAAAGGTGGTCAGTT	*Sca*I
BDH3UF	GGGGTACCCTCCTTTCCATACCGCAATC	*Kpn*I
BDH3UR	TCATACCTGCGGGACATTTGCGAGGTATTCGTGCAGGTC	
BDH3DF	GACCTGCACGAATACCTCGCAAATGTCCCGCAGGTATGA	
BDH3DR	GTGAGTACTCCCGCCTTCTATGAGTGG	*Sca*I
GDHUF	GGGGTACCTCGCTCATGGAAGGGTTAGT	*Kpn*I
GDHUR	ATGTTGTGGATGGTTTCGCCTCGGTGGAAGCAATGGTGGG	
GDHDF	CCCACCATTGCTTCCACCGAGGCGAAACCATCCACAACAT	
GDHDR	GTGAGTACTCCCTTGATTGTTGATCCTAT	*Sca*I

**Table 4:** Bacterial strains and plasmids used in this study.

Strain or plasmid	Relevant genotype and description	Reference or source
Strains		
*E. coli* DH5α	Host of plasmid for cloning	Lab stock
*E. coli* BL21(DE3)	Host of plasmid for expression, F-, ompT, hsdSB(rB-mB-), gal(λ c I 857, ind1, Sam7, nin5, lacUV-T7 gene1), dcm(DE3)	Lab stock
*Serratia* sp. T241	2,3-BD producer, wild-type	Lab isolation
*Serratia* sp. T241 Δbdh1	*bdh1* gene deletion mutant of *Serratia* sp. T241	This study
*Serratia* sp. T241 Δbdh2	*bdh2* gene deletion mutant of *Serratia* sp. T241	This study
*Serratia* sp. T241 Δbdh3	*bdh3* gene deletion mutant of *Serratia* sp. T241	This study
*Serratia* sp. T241 Δgdh	*gdh* gene deletion mutant of *Serratia* sp. T241	This study
*E. coli* S17-1 λ_pir_	*recA pro hsdR* RP4-2-Tc::Mu-Km::Tn7, conjugative strain able to host λ-pir-dependent plasmids	Lab stock
Plasmids		
pET28a	Expression vector, Km^R^	Novagen
pET-*bdh1*	pET28a carries *bdh1* gene	This study
pET-*bdh2*	pET28a carries *bdh2* gene	This study
pET-*bdh3*	pET28a carries *bdh3* gene	This study
pET-*gdh*	pET28a carries *gdh* gene	This study
pET-*budRAB*	pET28a carries *budRAB* genes	This study
pET-*budRAB-bdh1*	pET28a carries *budRAB* and *bdh1* genes	This study
pET-*budRAB-bdh2*	pET28a carries *budRAB* and *bdh2* genes	This study
pET-*budRAB-bdh3*	pET28a carries *budRAB* and *bdh3* genes	This study
pET-*budRAB-gdh*	pET28a carries *budRAB* and *gdh* genes	This study
pUTKm1	Ap^R^ Km^R^ *ori*R6K *ori*TRP4	Lab stock
pUT-*bdh1*	Plasmid used to delete *bdh1* gene	This study
pUT-*bdh2*	Plasmid used to delete *bdh2* gene	This study
pUT-*bdh3*	Plasmid used to delete *bdh3* gene	This study
pUT-*gdh*	Plasmid used to delete *gdh* gene	This study
