# Supplementary material for: Mechanism of 2,3-butanediol stereoisomers formation in a newly isolated Serratia sp. T241
Source: Sci Rep. 2016 Jan 12;6:19257. doi: 10.1038/srep19257 (PMC4709696; doi:10.1038/srep19257)
Supplement: Supplementary Information [file srep19257-s1.pdf]

Supplementary information for

Mechanism of 2,3-butanediol stereoisomers formation in a newly isolated *Serratia* sp. T241

Liaoyuan Zhang[1], Zewang Guo[1], Jiebo Chen[1], Quanming Xu[1], Hui Lin[1], Kaihui Hu[1], Xiong Guan[1]*, Yaling Shen[2]*

[1]Key Laboratory of Biopesticide and Chemical Biology, Ministry of Education, College of Life Science, Fujian Agriculture and Forestry University, FuZhou, Fujian province, 350002, PR China

[2]State Key Laboratory of Bioreactor Engineering, New World Institute of Biotechnology, East China University of Science and Technology, Shanghai, 200237, PR China

*Corresponding author: Key Laboratory of Biopesticide and Chemical Biology, Fujian Agriculture and Forestry University, Ministry of Education, FuZhou, Fujian Province, 350002, PR China Tel.: +86-591-83789492; Fax: +86-591-83789121;

E-mail: zliaoyuan@126.com, ylshen@ecust.edu.cn

Figure S1: Expression and purification of BDH1, BDH2, BDH3 and GDH enzymes from *Serraita* sp. T241. M, marker; 1, 5 and 11, *E. coli* harboring pET28a; 2, *E. coli* harboring pET28a-BDH1; 3, Crude enzyme of BDH1; 4, Purified enzyme of BDH1; 6, *E. coli* harboring pET28a-BDH2; 7, Crude enzyme of BDH2; 8, Purified enzyme of BDH2; 9, *E. coli* harboring pET28-BDH3; 10, Purified enzyme of BDH3; 12, *E. coli* harboring pET28a-GDH; 13, Crude enzyme of GDH; 14, Purified enzyme of GDH.

Figure S2: Chiral-column GC analysis of the substrates and products in the reduction and oxidation reactions catalyzed by BDH1, BDH2, BDH3 and GDH enzymes from *Serratia* sp. T241. BDH1 and BDH2, (A) Profile of mixture of standard chemicals; (B) The product from DA; (C) The product from (3*S*/3*R*)-AC; (D) The product from (2*S*,3*S*)-BD; (E) The product from *meso*-2,3-BD. BDH3 and GDH, (A) Profile of mixture of standard chemicals; (B) The product from DA; (C) The product from (3*S*/3*R*)-AC; (D) The product from (2*R*,3*R*)-BD; (E) The product from *meso*-2,3-BD.

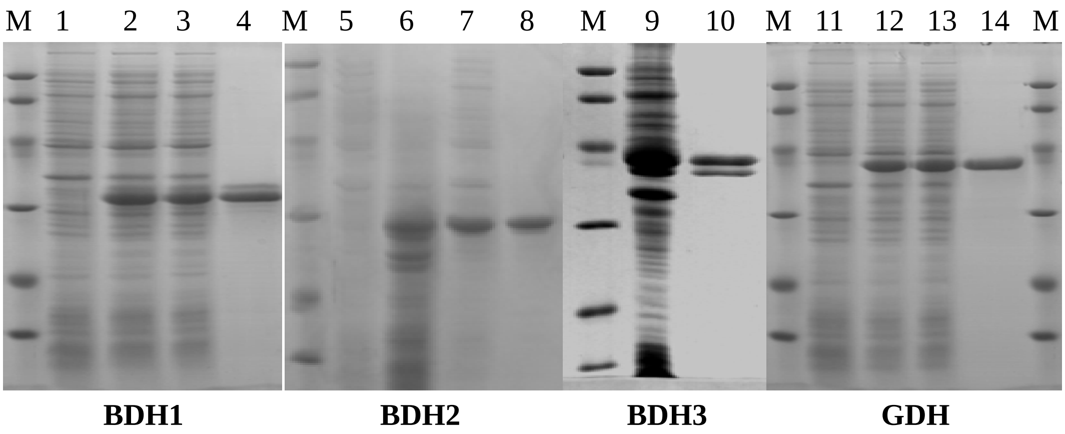
M 1 2 3 4 M 5 6 7 8 M 9 10 M 11 12 13 14 M
BDH1
BDH2
BDH3
GDH

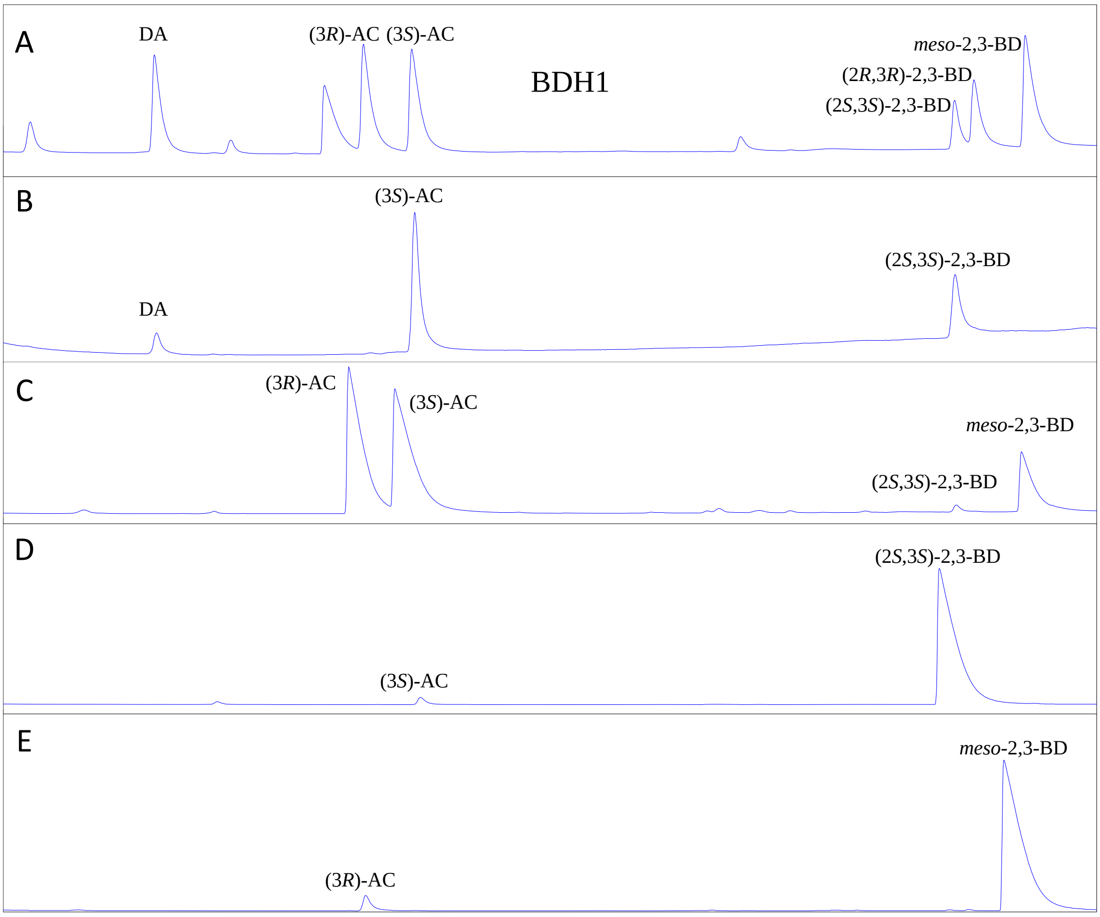
A
DA
(3*R*)-AC (3*S*)-AC
BDH1
meso-2,3-BD
(2*R*,3*R*)-2,3-BD
(2*S*,3*S*)-2,3-BD
B
(3*S*)-AC
DA
(2*S*,3*S*)-2,3-BD
C
(3*R*)-AC
(3*S*)-AC
meso-2,3-BD
(2*S*,3*S*)-2,3-BD
D
(2*S*,3*S*)-2,3-BD
(3*S*)-AC
E
meso-2,3-BD
(3*R*)-AC

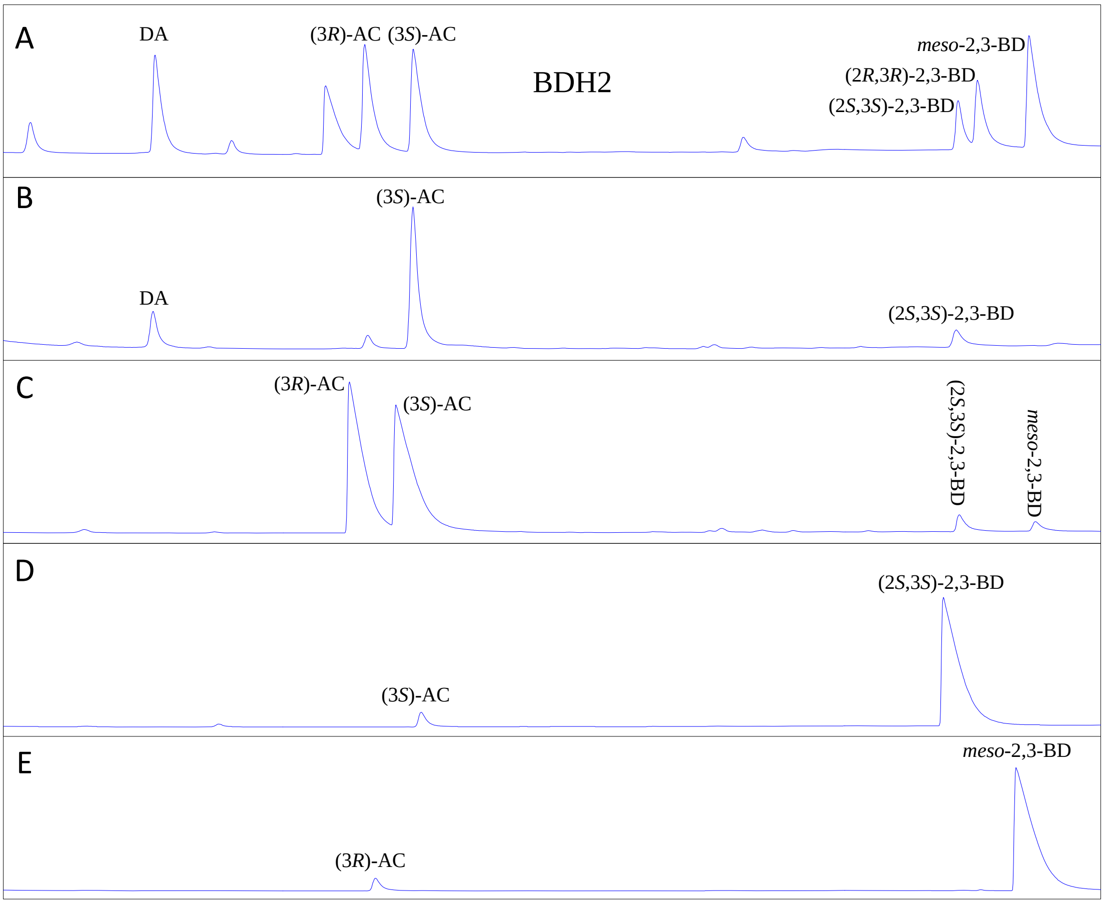
A
DA
(3R)-AC
(3S)-AC
BDH2
meso-2,3-BD
(2R,3R)-2,3-BD
(2S,3S)-2,3-BD
B
(3S)-AC
DA
(2S,3S)-2,3-BD
C
(3R)-AC
(3S)-AC
(2S,3S)-2,3-BD
meso-2,3-BD
D
(2S,3S)-2,3-BD
(3S)-AC
E
meso-2,3-BD
(3R)-AC

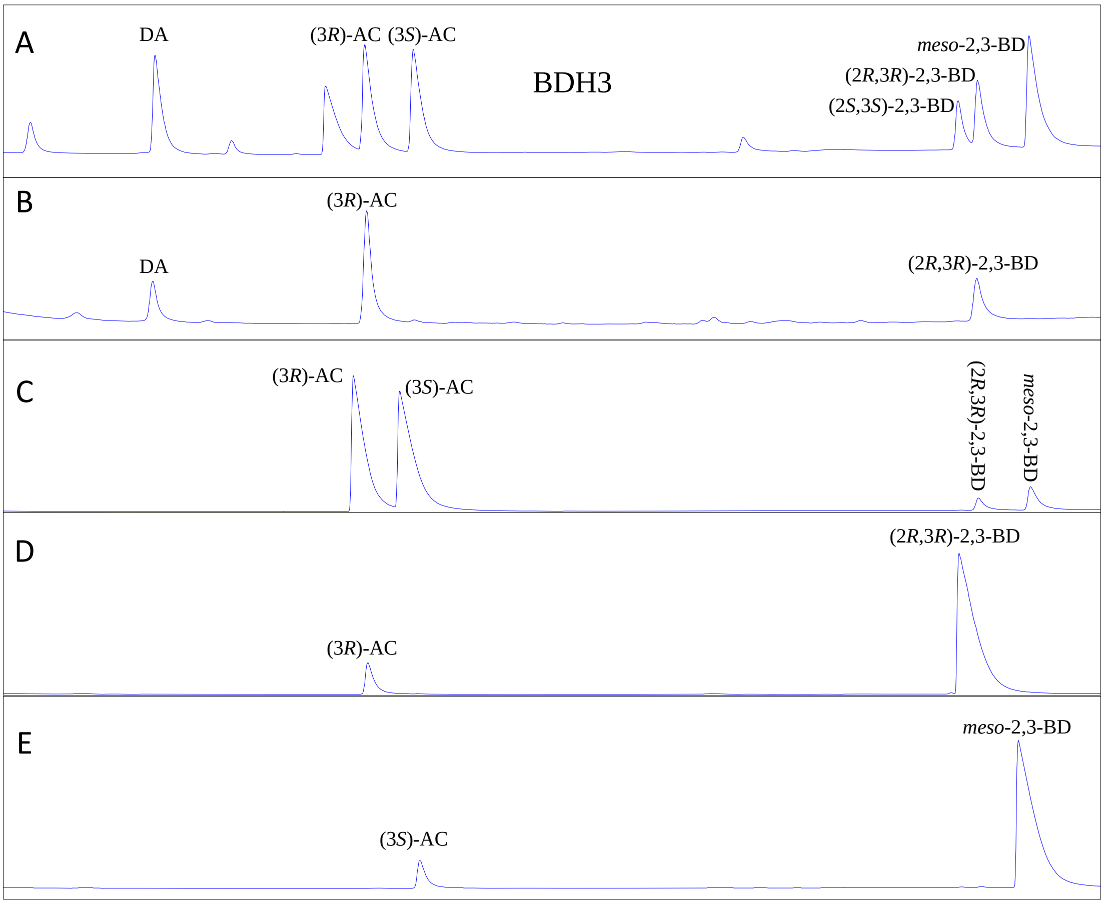
A
DA
(3*R*)-AC
(3*S*)-AC
BDH3
meso-2,3-BD
(2*R*,3*R*)-2,3-BD
(2*S*,3*S*)-2,3-BD
B
(3*R*)-AC
DA
(2*R*,3*R*)-2,3-BD
C
(3*R*)-AC
(3*S*)-AC
(2*R*,3*R*)-2,3-BD
meso-2,3-BD
D
(2*R*,3*R*)-2,3-BD
(3*R*)-AC
E
meso-2,3-BD
(3*S*)-AC

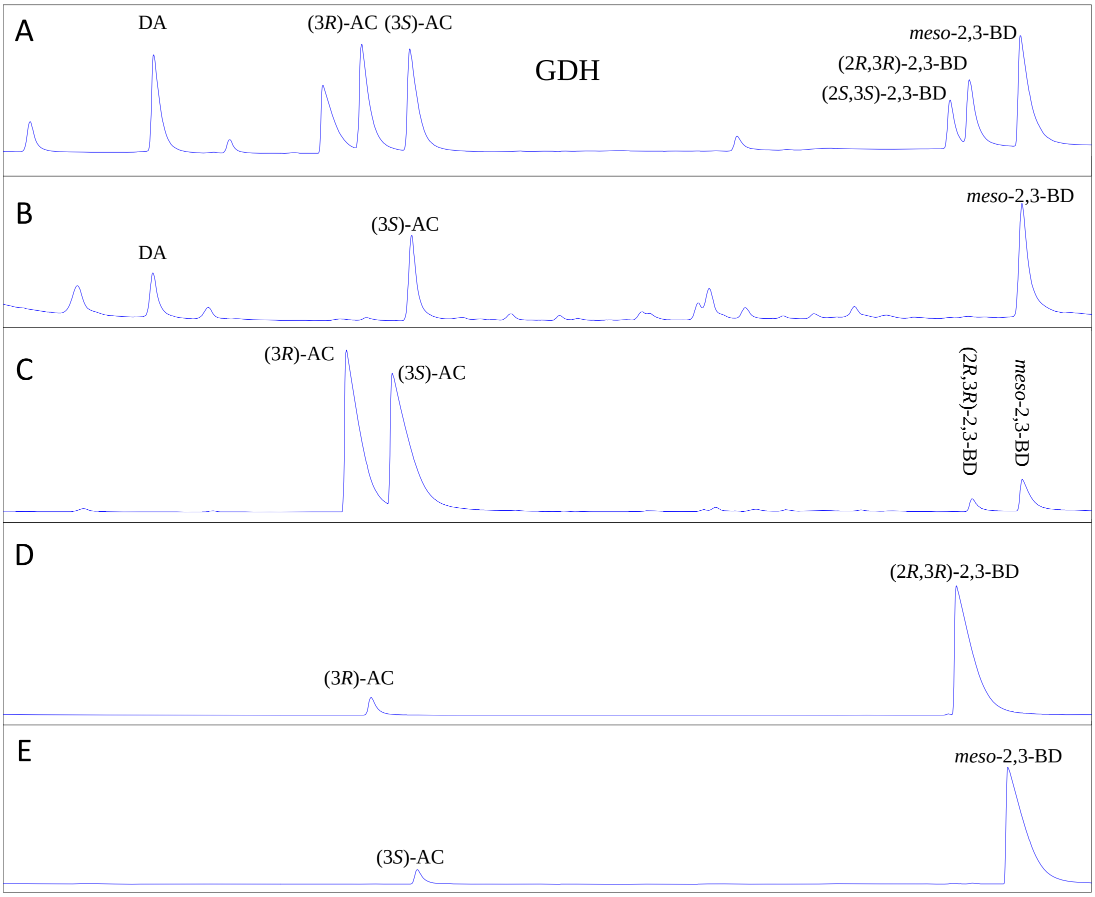
A
DA
(3R)-AC (3S)-AC
GDH
meso-2,3-BD
(2R,3R)-2,3-BD
(2S,3S)-2,3-BD
B
DA
(3S)-AC
meso-2,3-BD
C
(3R)-AC
(3S)-AC
(2R,3R)-2,3-BD
meso-2,3-BD
D
(2R,3R)-2,3-BD
(3R)-AC
E
meso-2,3-BD
(3S)-AC
